# Identification and treatment of offenders with attention-deficit/hyperactivity disorder in the prison population: a practical approach based upon expert consensus

**DOI:** 10.1186/s12888-018-1858-9

**Published:** 2018-09-04

**Authors:** Susan Young, Gisli Gudjonsson, Prathiba Chitsabesan, Bill Colley, Emad Farrag, Andrew Forrester, Jack Hollingdale, Keira Kim, Alexandra Lewis, Sarah Maginn, Peter Mason, Sarah Ryan, Jade Smith, Emma Woodhouse, Philip Asherson

**Affiliations:** 1 Psychology Services Limited, Croydon, UK; 2 Institute of Psychiatry, Psychology and Neuroscience, Department of Psychology, King’s College London, London, UK; 3 Offender Health Research Network, Division of Psychology and Mental Health and Division of Medicine and Health, University of Manchester, Manchester, UK; 4 CLC Consultancy, Dunkeld, Scotland, UK; 5 Sussex Partnership NHS Foundation Trust, Children & Young People’s Service, Tunbridge Wells Kent, UK; 6 Forensic Psychiatry and Offender Health Research Network, Division of Psychology and Mental Health and Division of Medicine and Health, University of Manchester, Manchester, UK; 7 Division of Psychology and Language Science, University College London, London, UK; 8 Independent Medical Writer, San Diego, California USA; 9 Barnet Enfield & Haringey Mental Health NHS Trust, HMP/YOI Feltham, Feltham, UK; 10 Health & Justice Central Team, NHS, London, UK; 11 Kent Prisons, Oxleas NHS Foundation Trust, Dartford, UK; 12 Cheshire & Wirral Partnership NHS Foundation Trust, The Stein Centre, St Catherine’s Hospital, Birkenhead, UK; 13 Tees, Esk & Wear Valley NHS Foundation Trust, HMP Frankland, Durham, UK; 14 Humber NHS Foundation Trust, East Yorkshire, London, UK; 15 Institute of Psychiatry, Psychology, and Neuroscience, Department of Forensic and Neurodevelopmental Sciences, King’s College London, London, UK; 16 South London & Maudsley NHS Foundation Trust, National Adult Outpatient Neurodevelopmental Clinic, and National Autism Unit, London, UK; 17 Institute of Psychiatry, Psychology, and Neuroscience, King’s College London, London, UK

**Keywords:** Attention-deficit/hyperactivity disorder (ADHD), Prison population, Identification, Treatment, Interventions, Consensus, UKAP

## Abstract

**Background:**

Around 25% of prisoners meet diagnostic criteria for attention-deficit/hyperactivity disorder (ADHD). Because ADHD is associated with increased recidivism and other functional and behavioural problems, appropriate diagnosis and treatment can be a critical intervention to improve outcomes. While ADHD is a treatable condition, best managed by a combination of medication and psychological treatments, among individuals in the criminal justice system ADHD remains both mis- and under-diagnosed and consequently inadequately treated. We aimed to identify barriers within the prison system that prevent appropriate intervention, and provide a practical approach to identify and treat incarcerated offenders with ADHD.

**Methods:**

The United Kingdom ADHD Partnership hosted a consensus meeting to discuss practical interventions for youth (< 18 years) and adult (≥18 years) offenders with ADHD. Experts at the meeting addressed prisoners’ needs for effective identification, treatment, and multiagency liaison, and considered the requirement of different approaches based on age or gender.

**Results:**

The authors developed a consensus statement that offers practical advice to anyone working with prison populations. We identified specific barriers within the prison and criminal justice system such as the lack of adequate: staff and offender awareness of ADHD symptoms and treatments; trained mental health staff; use of appropriate screening and diagnostic tools; appropriate multimodal interventions; care management; supportive services; multiagency liaison; and preparation for prison release. Through discussion, a consensus was reached regarding prisoners’ needs, effective identification, treatment and multiagency liaison and considered how this may differ for age and gender.

**Conclusions:**

This practical approach based upon expert consensus will inform effective identification and treatment of offenders with ADHD. Appropriate intervention is expected to have a positive impact on the offender and society and lead to increased productivity, decreased resource utilization, and most importantly reduced rates of re-offending. Research is still needed, however, to identify optimal clinical operating models and to monitor their implementation and measure their success. Furthermore, government support will likely be required to effect change in criminal justice and mental health service policies.

**Electronic supplementary material:**

The online version of this article (10.1186/s12888-018-1858-9) contains supplementary material, which is available to authorized users.

## Background

Effective identification and treatment of offenders with attention-deficit/hyperactivity disorder (ADHD) in the prison population is likely to have a positive impact on the offender and society. ADHD is associated with early age criminality [1], a high rate of recidivism [2], and a two to three-fold increased risk of later arrest, conviction, and imprisonment [1]. While ADHD is a treatable condition best managed by a combination of appropriate medication and psychological treatments [3], among individuals in the criminal justice system ADHD remains both mis- and under-diagnosed [4–6] and consequently inadequately treated.

ADHD is characterized by symptoms of pervasive and impairing inattention and/or hyperactivity and impulsivity [7] that starts during childhood or early adolescence and persists in around half of individuals into adulthood [8], where it is associated with significant personal, social, and occupational problems [9]. Compared with inmates without ADHD, inmates with ADHD symptoms demonstrate a high frequency and severity of functional impairment that worsen in proportion to the severity of their ADHD symptoms [4, 5].

Worldwide prevalence rates estimate that 5.3% of children and 2.5% of adults meet diagnotic criteria for ADHD [10, 11]. Meta-analyses of 42 prisons, based on international data derived from symptom-based clinical diagnostic interviews, indicated that 25.5% of the prison population overall met diagnostic criteria for ADHD [12]. Compared with the worldwide prevalance of ADHD, this is a five-fold increase among youth prisoners (< 18 years) and a ten-fold increase among adult prisoners (≥18 years) [12].

Among incarcerated adults with ADHD, there is an increased risk of associated coexisting psychopathology [13] that often confounds and influences treatment options. Given the high risk of co-morbid mood disorders among youth and adult offenders with ADHD [5], paired with their high risk of self-harm or suicide in the first weeks of prison reception [14], it is imperative to identify prisoners at risk of aggression, violence, self-harm, and suicide who might benefit from treatment for ADHD. It is also important to identify prisoners with ADHD with co-morbid substance use disorders, so that they may undergo detoxification treatment in prison before receiving treatment for ADHD. Adults with undiagnosed ADHD treated for other mental health disorders such as major depression, anxiety, bipolar, and/or personality disorders, have poor clinical and functional outcomes if ADHD goes untreated [5]. When appropriately diagnosed and treated for ADHD, there is likely improved ADHD symptom control, emotional lability, and overall functioning. Furthermore, outcome studies indicate reduced rates of transport accidents, criminality, and suicidal behaviour during periods of treatment for ADHD [5, 15–17].

Although there is a large evidence base for ADHD treatments for individuals in the community, similar evidence is limited for those in the prison population. A literature review of non-offender populations with ADHD reported that the combination of pharmacological and non-pharmacological treatment was most consistently associated with improved long-term outcomes and large effect sizes [3]. An Icelandic study of non-offender adults with ADHD reported that those who received the multi-modal treatment of ADHD medication plus the ADHD version of Reasoning and Rehabilitation 2 for Youths and Adults (R&R2ADHD) [18], experienced a significant reduction in ADHD and co-morbid symptoms and demonstrated improved functional outcomes [19–21]. A Swedish national database study of released prisoners reported that rates of violent reoffending were reduced by 42% during periods when they were receiving antipsychotics, psychostimulants, and/or drugs for addictive disorders, compared to periods in which they were not receiving medication [22]. Another Swedish database study reported that among those treated for ADHD, criminal conviction rates were reduced by 32% in men and 41% in women over a 3 year period [23].

As of October 2015 the World Prison Population List conservatively estimated that up to 11 million people were held in penal institutions throughout the world [24]. With around a quarter of prisoners worldwide meeting diagnostic criteria for ADHD, we estimate that 2.8 million prisoners have ADHD.

Given the large population of prisoners with ADHD combined with their increased risk of recidivism, appropriate intervention is crucial. By expanding upon the consensus of the United Kingdom Adult ADHD network (UKAAN) on the identification and management of offenders with ADHD [6], we aimed to identify existing barriers within the prison system that prevent appropriate intervention, and provide a practical approach to effectively identify and treat incarcerated offenders with ADHD.

## Methods

The United Kingdom ADHD Partnership (UKAP; www.UKADHD.com) hosted a meeting in November 2016, where researchers, prison staff, clinicians, and patient representatives with expertise in offender mental health and ADHD, convened to discuss identification and treatment of youth and adult offenders with ADHD in the prison population. Each author attended the meeting. The authors represent a multidisciplinary group including both prescribing and non-prescribing clinical and academic experts, with extensive experience working with individuals with ADHD, including prisoners (for further details see Authors’ Information subsection of the Declarations section). The meeting included presentations with electronic slides, discussions, and group work. Presentations and discussions were recorded and later transcribed.

The meeting commenced with four presentations:

- The Facts: What We Know from Empirical Data
- Needs, Problems, and Obstacles when Assessing and Treating ADHD in a Young Offender Institution
- Needs, Problems, and Obstacles when Assessing and Treating ADHD in an Adult Prison
- Beyond the Gates: Needs, Multiagency Liaison, and the Care Pathway

Following the presentations, all attendees separated into three small groups. Each group was tasked with providing practical solutions relevant to their assigned topic. The methodological orientation that underpinned the focus of the discussion groups was phenomenological, using the empirical research base and their clinical experience. Group leaders facilitated the small-group discussions while scribes took notes and summarized their groups’ answers to the following topical questions:

1. Identification and Assessment
  How do we identify ADHD among youth offenders? Among adult offenders? What are the screening tools?
  What should trigger additional assessments and referrals? And to whom?
  What should these assessments involve for youth offenders? For adult offenders?
  Are there significant gender differences to take into account?
2. Interventions and Treatment
  What are the appropriate pharmacological treatments for youth offenders? For adult offenders?
  What are the appropriate non-pharmacological treatments for youth offenders? For adult offenders?
  What is the evidence base for these treatments?
  Are there significant gender differences to take into account?
3. Care Management and Multiagency Liaison
  What agencies need to be involved in developing a care plan for youth offenders? For adult offenders?
  What might trigger multiagency liaison?
  What should be considered when providing support to families and carers?
  What kind of educational, behavioural, and/or socialization support should be established to meet the needs of this population?
  Are there significant gender differences to take into account?

Following the small-group work, all attendees re-assembled together. The leaders then presented their findings in a feedback session to all the attendees for another round of discussion and debate, until a final consensus was reached. The medical writer consolidated the meeting transcription, electronic slide presentations, and small-group notes into the manuscript. Lastly, the meeting transcription and manuscript were circulated to all authors for review to ensure agreement. The consensus reported here reflects the views of the authors based on their experience and is supported by published research; and aimed to provide practical guidance to health care professionals working with prisoners with ADHD.

## Results

The authors successfully came to a consensus on a practical approach to identify and treat prisoners with ADHD. While our approach draws primarily from our experiences in the UK, we believe it can be easily adapted for use in other countries.

## Identification and assessment

The identification and accurate diagnosis (including confirmation of a previous diagnosis) of ADHD among prisoners is a process reliant on the availability of medical records, mental health clinicians trained to conduct interview assessments for ADHD, prison staff trained to recognise potential patients with ADHD, and suitable screening and diagnostic instruments. It will be necessary to use identification and assessment tools that are specific to youth or adult offenders. According to best practice all new prisoners should receive an initial reception screen for mental health problems, including ADHD, followed by a comprehensive second screen shortly after their reception screen [25, 26].

### Identifying prisoners with ADHD

While some prisoners present with a history of ADHD diagnosed during childhood, others present with no history of ADHD, or alternative diagnoses such as specific learning difficulties (dyslexia, dyspraxia) or conduct problems. It is therefore important to review the medical history and confirm the presence of other common mental health and neurodevelopmental disorders that may overlap with ADHD — highlighting the need for a careful diagnostic assessment. In our experience, many offenders who had a prior diagnosis of ADHD were untreated or failed to adhere to their treatment programme. In making a new diagnosis, there are specific indicators among the prison population that, in our experience, suggest the presence of ADHD and are as follows:

- Symptoms of inattention, which are often missed among offenders of both genders
- Symptoms of impulsivity, emotional dysregulation, and poor self-control, which are especially important to recognize as the prisoner may be at risk of aggression and violence towards others, or self-harm and suicide
- A history of educational failure, school expulsion, inability to work, driving offences, and impulsive aggression, and/or
- A history of chronic mental health problems or a history of failed treatment programmes for conditions such as mood disorders, anxiety, depression, post-traumatic stress disorder, emotional instability, self-harm, and/or borderline personality disorder.

We have observed that assessors commonly view hyperactivity as a ‘male’ ADHD symptom and inattention as a ‘female’ ADHD symptom. This bias may interfere with making an accurate diagnosis of ADHD in males who lack overt hyperactivity and in females who display hyperactive behaviour. Diagnosing ADHD in offenders can be additionally challenging because it is often complicated by the high frequency of co-occurring conditions. In addition to common disorders seen in the prison population such as anxiety, depression, post-traumatic stress disorders, substance abuse and self-harm, borderline personality disorders are commonly found among female offenders [27, 28], and conduct and antisocial personality disorder among males; all potentially masking the diagnosis of ADHD. Consequently, further training is often required to support the accurate identification of ADHD symptoms and distinguish these from other disorders.

### Prison staff ADHD awareness training

Currently there is very limited training about ADHD for prison staff. Raising awareness of ADHD was however considered to be essential by the consensus group. ADHD awareness training is likely to reshape misconceptions or stereotypes of ADHD and may improve the outcomes of offenders with ADHD within the criminal justice system. It is important to understand that although ADHD is a pervasive condition that persists into adulthood in around half of cases, it is treatable at all ages. Prison officers, clinicians, educators, therapists, and mentors therefore should be trained to recognize the signs and symptoms of ADHD and further educated on available treatments and expected outcomes.

ADHD awareness training should ideally raise the visibility of the disorder. Increased understanding and recognition of ADHD will likely help prison staff to better manage offenders with ADHD presenting with difficult behaviours that were previously unattributed to the disorder [2]. While any member of prison staff can refer a prisoner for a mental health assessment at any time, referrals are dependent on their vigilance and ability to make appropriate observations.

Delivery of ADHD awareness training (alongside training for mental health more generally) in the prison setting may have logistical challenges, such as lockdown requirements to allow staff to attend workshops. However, training in mental health issues, including ADHD, is essential for this high-risk population and needs to be addressed at each institution.

### Screening for youth offenders

#### Primary screen

As is the case for many prisons, all youth offenders are subject to a reception screen upon admission. Screening preferences and practices vary greatly between institutions world-wide, and while no evident gold standard method exists, healthcare standards offer guidance for best practices for youth in secure settings [29]. Nurses with mental health experience usually administer the primary screen to assess the overall physical and mental health of the offenders.

The National Health Service England (NHSE) mandates using the comprehensive health assessment tool (CHAT) in youth offender institutions throughout England and Wales. Because of the NHSE requirements, we recommend using the CHAT as the primary screen in all youth offender institutions. The CHAT is a validated semi-structured interview designed to screen for health issues among all young offenders admitted to a secure facility [30]. CHAT is divided into four sections covering: physical health, mental health, substance abuse, and neurodisability. It is available in electronic format; however, the mental health section takes approximately one hour to complete.

Questions pertaining to ADHD symptoms are included in the mental health section of the CHAT, but because they focus on externalizing rather than internalizing symptoms the assessors will need to take extra care in considering all symptoms, including the inattention symptoms of ADHD. While we acknowledge the poor specificity of CHAT, it is a sensitive tool for detecting mental health problems that include ADHD. It is therefore important to emphasize that the CHAT should be used as a primary screen to flag potential mental health issues before going on to more detailed assessments.

If any chronic or serious mental health issue is suspected, then the offender should be referred for a secondary screen involving a more comprehensive assessment by a multidisciplinary mental health team including nurses, psychologists, and psychiatrists who are specially trained to recognize ADHD as well as other mental health conditions commonly seen in young offenders.

#### Secondary screen and clinical diagnosis

At the time of clinical assessment, we recommend that a trained clinician administer a standard validated rating scale such as the Swanson, Nolan, and Pelham teacher and parent rating scale (SNAP-IV) [31] and/or the Conners’ comprehensive behaviour rating scale (Conners’ CBRS) [32] to assist in making the diagnosis and to gather necessary collateral information. A diagnosis of ADHD usually requires supporting evidence from teacher, therapist, employer, or parent reports. In cases where screens indicate a possible ADHD diagnosis, we recommend that clinicians always conduct a full diagnostic interview for ADHD, as well as carefully assess for commonly occurring co-morbid conditions such as drug abuse, personality disorders, emotional problems, and learning difficulties. The ADHD Child Evaluation (ACE) [33] is one such tool used for assessing ADHD symptoms, possible co-morbid problems, and associated impairments. The ACE is a semi-structured diagnostic interview providing either DSM-5 or ICD-10 [7] criteria and is available in 19 languages free of charge in paper form. Those who are symptomatic on screening, but have a confirmed pre-existing diagnosis of ADHD may not require a diagnostic interview. This will largely depend on how long ago the diagnosis was made and is a matter of clinical judgment.

### Screening for adult offenders

#### Primary screen

In adult prisons offenders are subject to a reception screen upon admission, to review their overall physical and mental health. In most institutions, however, the mental health section of the primary screen for adult offenders does not include questions pertaining to ADHD symptoms. For example, the screening tool Health & Wellbeing Needs Assessment Toolkit (HWBNA) published by the Public Health England Health & Justice Team, to be used in all 118 adult English Prisons, has no mention of ADHD. Screening preferences and practices vary greatly between adult institutions world-wide and while no evident gold standard method exists, several valid tools are available [25].

Some prisons have adopted the short screening version of the Adult ADHD Self Rating Scale (ASRS), [34] yet in our experience, because of the wording of the questions, this is less suitable for prison populations. We identify this as problematic and therefore suggest using the brief version of the Barkley Adult ADHD rating scale (B-BAARS) as part of the primary mental health screen. The B-BAARS is a short, six-item screen with excellent specificity and sensitivity for predicting a diagnosis of ADHD in the offender population, and is available free of charge [33, 35].

Ideally, nurses with mental health experience should administer the primary screen in adult institutions as this increases the validity of the screens by ensuring questions are understood and properly rated by the offenders [36]; however, this is usually not the case. Nurses in adult prisons and police stations tend to be physical health nurses without mental health experience. This may cause difficulties with identifying ADHD symptoms, as well as other mental health disorders, in the primary screen and may prevent triggering a secondary screen among adult offenders. Because of this, we recommend employing nurses with mental health experience throughout the criminal justice system offender pathway to adequately assess the overall physical and mental health of adult offenders.

If any co-morbid mental health disorder is suspected, then the offender should be referred for a secondary screen involving a more comprehensive assessment by a multidisciplinary mental health team including nurses, psychologists, and psychiatrists who are specially trained to recognize ADHD as well as other adult mental health conditions.

#### Secondary screen and clinical diagnosis

In cases where a B-BAARS score or results from another screen indicate a likely ADHD diagnosis, we recommend a clinician trained in the assessment of ADHD conduct a full diagnostic interview. This may include the full 18-item version of the BAARS to ascertain the severity of ADHD. Whenever possible, the interview should be supported by collateral reports (e.g., parent, therapist, and previous medical history) to gain a comprehensive description of their symptoms and impairments across the lifespan. While informant reports are not always required, they can be particularly useful as offenders with ADHD often have very poor recollections of their childhood behaviour and tend to minimise impairments arising from ADHD. Extra care is needed when evaluating all of the offenders’ symptoms to avoid focusing only on externalising behaviours.

We have identified three comprehensive semi-structured diagnostic interview tools that are suitable for adult prison populations: the Conners’ Adult ADHD Diagnostic Interview for DSM-IV (CAADID) [37], the Diagnostic Interview for ADHD in adults (DIVA-2) [38, 39], and the ACE+ (ACE for adults) [40]. While the CAADID and DIVA-2 are the more established methods, the ACE+ has an advantage because it includes a section that considers coexisting conditions and whether they are co-morbid or reflect a differential diagnosis. For this reason, the ACE+ may be preferred when establishing the diagnosis of ADHD in the presence of co-morbid disorders. The DIVA-2 is widely used throughout Europe and should be used in conjunction with a systematic assessment of co-morbidity because it does not include a prompt to evaluate the presence of common co-morbid conditions. Both the ACE+ and DIVA-2 are available in many languages other than English and are free of charge in paper form.

Each of these interviews provides age-appropriate examples of each of the ADHD symptoms that can be applied when evaluating adults. Relevant descriptions of adult ADHD symptoms include: internal restlessness; excessive mind wandering that interferes with tasks such as reading, writing and listening to TV or conversations; getting bored quickly then losing ability to focus; and feelings of irritability and impatience when waiting in queues. Clinicians also need to be aware that symptoms of emotional dysregulation such as frequent inappropriate levels of irritability, frustration, and anger (while not part of the formal diagnostic criteria) can be used to support the diagnosis [7].

While continuous performance tests (CPT) perform reasonably well at discriminating between people with ADHD from non-psychiatric controls, and may contribute to the diagnostic assessment, they are far less able to discriminate ADHD from other common psychiatric disorders seen in prison populations [38]. Given these validity issues and the logistical problems of bringing information technology equipment into prisons, we cannot recommend their use in prison populations.

## Interventions and treatment

Providing appropriate pharmacological and non-pharmacological interventions in prison populations is a process reliant on prison staff and offender self-awareness of ADHD symptoms and treatments, the availability of trained mental health clinicians, and the administration of specifically targeted multimodal treatments. Although female offenders often present with a more complex profile due to pregnancy, motherhood, and high levels of co-morbidity [13], all pharmacological and non-pharmacological treatments can be administered irrespective of age and gender. During pregnancy, ADHD pharmacological treatments can be used, but should be restricted to cases where treatment is required to reduce significant distress and behavioural problems, where the potential risks of no treatment outweigh risks of treatment [41].

**Fig. 1:**
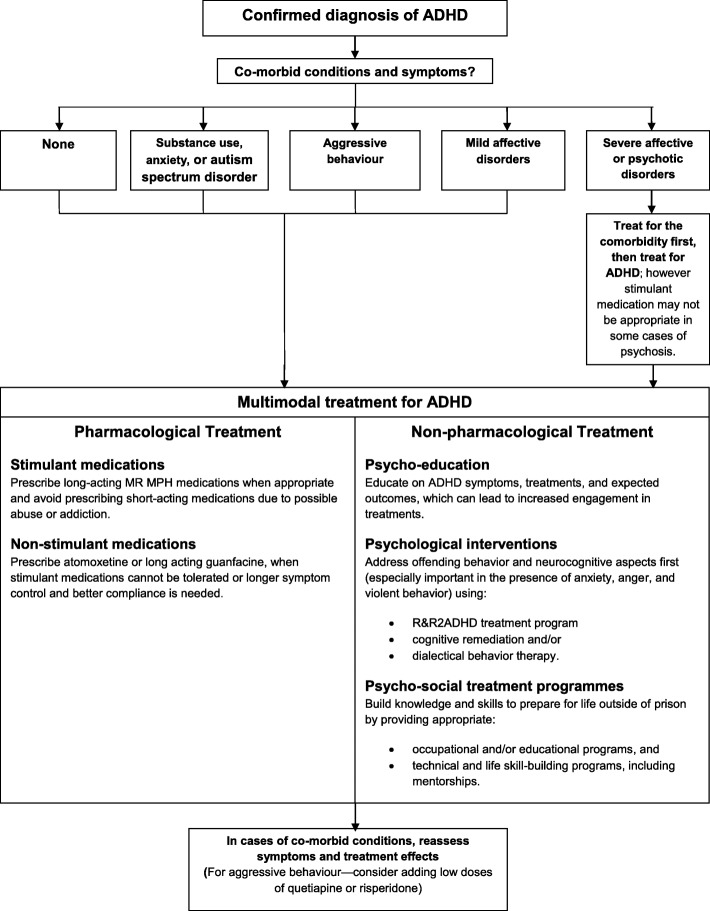
Multimodal Treatment for Incarcerated Offenders with ADHD

It will be necessary to educate offenders on the efficacy of multimodal treatments and expected outcomes and to obtain informed consent for permission to treat. Following a new or confirmed previous diagnosis of ADHD and a careful evaluation of possible co-morbid conditions, we recommended the following multi-modal treatment for incarcerated offenders, as summarized in Fig. 1.

We have observed that staff support in pharmacological treatment and involvement in psychological treatment programmes has a positive and direct impact on offender adherence to and engagement in the prescribed treatment plan. Staff lack of knowledge about ADHD can interfere with medication administration and offender engagement in psychological treatment programmes. Conversely, staff awareness of ADHD symptoms, and observation of positive treatment effects, usually increases engagement with and support for the treatment process, and makes it much more likely they become involved in the delivery of offenders’ psychological treatments.

According to principle and law, punishment for criminal offence is the loss of liberty, and prisoners’ human rights are fully protected unless restrictions are unavoidably and demonstrably ‘necessitated by the fact of incarceration’ [42]. The Mandela Rules expressly state the fundamental prohibitions on torture and inhumane treatment, and emphasize that imprisonment is itself the punishment and should not carry additional ‘pains’ [42]. Solitary confinement is arguably considered to be an additional ‘pain’, with especially significant adverse effects for people with serious mental illness; it has negative psychological consequences and restricts the inmates access to mental health services [43, 44]. Although ADHD is often un-recognized as a serious mental illness, we contend that severe cases should be recognized as such, and particularly when ADHD related behaviours are severe enough to lead to isolation of sentenced prisoners. We have observed that solitary confinement exacerbates prisoners’ ADHD symptoms [45], and recommend increasing efforts to ensure that offenders with ADHD are prevented from receiving this punishment. The same argument can be applied to severe cases of other mental health disorders, so it not unique to ADHD. This may require prisons to reduce the use of solitary confinement for all prisoners and to ensure that it is only used as a last resort, for short periods of time to manage acute situations, and never as a punishment.

Given the high prevalence of neurodevelopmental disorders (traumatic brain injury, communication disorders, dyslexia, learning disabilities, autistic spectrum disorder, and ADHD) among young offenders [46], it is especially important to staff the prisons appropriately with educational psychologists and speech/language therapists to meet their needs adequately. Currently, prisoners with these needs are either inadequately supported or receive these necessary services only upon release.

### Pharmacological treatments for offenders

Treatment with ADHD medication is effective in reducing symptoms of inattention, hyperactivity, and impulsiveness [47] and is also reported to be associated with a significant reduction in violent reoffending (around 42%) on release from prison [22] and similarly in criminal convictions [23]. By reducing their ADHD symptoms, the offender is likely better equipped to engage in and benefit from psychological, educational, and occupational interventions. It is important for offenders and prison staff to understand that, although ADHD medication reduces ADHD symptoms, it does not cure the disorder, and concomitant non-pharmacological treatments are nearly always necessary to help the offender manage ADHD related problems and improve their behaviour.

Given the lack of sufficient high quality evidence from randomized-control trials for treatment among prisoners, it is important to emphasize that clinicians trained in the diagnosis and treatment of ADHD should administer ADHD medications, to carefully asses for risks of abuse potential and possible risks of exacerbating other co-morbid conditions. Medication can be administered to all offenders irrespective of age and gender, but type and dosage should be made on an individual basis, especially in the case of pregnancy.

With respect to psychoeducation, the offender needs to be educated on the benefits and side effects of pharmacological treatment and the implications of remaining untreated or discontinuing treatment. This education is not only necessary in obtaining informed consent to treat, but is important in engaging them in their own treatment. Such engagement will encourage offenders to take an active role in their treatment and can cause them to perceive some control over their situation, which can have an empowering effect. Offenders need to be given adequate opportunities to provide feedback on the medication’s effect and to titrate for therapeutic doses. Support during the early stage of treatment is critical in helping offenders take full advantage of the reductions in ADHD symptoms and in helping manage the medication's side effects.

We recommend prescribing stimulant medications first line because these have a quicker response than non-stimulants and a greater average effect size. Drugs with a high risk of abuse, such as immediate release preparations of methylphenidate (MPH) and dexamfetamine (DEX), should be avoided in prison populations due to the potential for abuse. Injected or insufflated (snorted) MPH and DEX cause a rapid release of dopamine that can give the user a ‘high’. This does not occur, however, when these medications are taken orally. The oral administration of therapeutic doses of MPH or DEX is therefore essential in reducing the abuse potential of stimulant medications [48].

We recommend prescribing long acting or modified release preparations of methylphenidate (MR MPH) that are difficult to take in any other way than by mouth (e.g. Concerta XL). Lisdexamfetamine (Elvanse) is a long acting preparation that has a unique advantage, because even if injected, the active drug is released slowly at a similar rate in to the brain as when taken by mouth. These extended release formulations are usually taken in the morning and give active control of symptoms for 8–14 h in most cases.

MR MPH, lisdexamfetamine, along with all other stimulants, are controlled substances and thus subject to strict dispensary logistics that often interfere with treatment compliance and efficacy. Restrictions on movements within the prison can limit the regular and timely administration of stimulant (and other) medications. In some cases, staff are needed to escort prisoners to the healthcare unit to receive medication, although in most cases prisoners can take themselves to a dispensary, or receive medication from a wheeled cart. Escorting not only over burdens the staff, but stigmatizes the offender and further complicates adherence. Although stimulants are controlled substances, they can usually be dispensed in the same manner as other non-stimulant medications that are not kept in possession, which improves adherence. The practice of dispensing drugs varies by prison, but non-stimulants are easier to dispense as there are less procedures for nursing staff to follow.

Preliminary results of a pilot study of Concerta XL in adult offenders with ADHD (CIAO) indicated a significant reduction in total critical incidents (assaults, fights, property damage, self-harm, drug use, and acts of disobedience) among prisoners in the UK who were treated for 12 weeks. In relation to dose, over half of the prisoners took 18-36 mg and only 4% took the maximum dose of 90 mg, indicating a lack of drug seeking behaviour with regard to Concerta XL in this population. This was in line with our clinical experience that suggests greater abuse potential for sedative antidepressants and antipsychotics than stimulants within the prison population. The findings from this study were successfully used to secure further funding the National Institute of Health Research (NIHR) for an ongoing randomised controlled trial in 200 young adult offenders following a similar study design. We anticipate that the reports from these studies will inform optimal medical treatment of ADHD in prisons and raise public awareness for the need for effective treatment of offenders with ADHD (unpublished report for pilot study available from PA).

In alignment with national guidelines [49], we recommend prescribing non-stimulants, such as atomoxetine in adolescents under 18 and adults, and/or long-acting guanfacine in adolescents under 18: when stimulants do not adequately treat symptoms or cause adverse effects, when a sustained 24-h effect is required, or there is clear drug seeking behaviour for stimulant medications (a rare event in our experience). In adults there is no data for the use of guanfacine, so atomoxetine is the non-stimulant of choice. Non-stimulants are easy to administer as they are not a controlled substance and would therefore bypass dispensary logistics and potentially improve treatment compliance. Although atomoxetine and long-acting guanfacine take several weeks to reach optimal effect, they have a significantly longer effect on symptom control over a 24-h period and can maintain their effect when individual doses are missed. They are particularly useful for patients who have a rapid return of severe ADHD symptoms once stimulant effects wear off during the day. Additionally, non-stimulants are the medication of choice for patients with a previous history of stimulant abuse.

#### Pharmacological treatments for offenders with co-morbid conditions

In the presence of co-morbid anxiety, autism spectrum disorder (ASD), aggressive behaviour, or mild affective symptoms, ADHD should usually be treated first, followed by a careful evaluation of the medication’s effect on the co-morbid symptoms. While adults with ADHD are reported to misuse drugs [50, 51], detoxification is provided by prison mental health services, and despite reports in the media about drug abuse in prison, there is no longer regular access to major drugs of abuse. Substance abuse is stabilised and under control in most cases in prison settings, so that diagnostic assessments and treatment for ADHD can proceed.

Symptoms commonly shared between ADHD and co-morbid disorders may be better managed with pharmacological treatments for ADHD rather than with pharmacological treatments for the co-morbid disorders themselves. For example, irritability and low mood symptoms secondary to ADHD are alleviated more effectively by ADHD medication than with antidepressants or antipsychotics. Similarly, we have observed that conditions such as post-traumatic stress disorder and borderline personality disorder sometimes improve following treatment of concurrent ADHD. Subsequent treatments for co-morbid disorders may be required and can be added one at a time to discriminate their effects. Conversely, in the presence of psychosis, bipolar disorder, and/or a clear depressive episode, ADHD should not be treated first. Care should be taken, however, to avoid mistaking the ADHD symptoms of emotional instability for the episodic mood changes of bipolar disorder or the chronic symptoms of a personality disorder.

In the case of co-morbid anxiety disorder, pharmacological treatment for the anxiety can be added if the stimulant exacerbates the anxiety. Alternatively, the stimulant can be discontinued and replaced by atomoxetine. In the case of co-morbid symptoms of aggressive behaviours, a low dose of quetiapine or risperidone may be added if the symptoms are not adequately treated by stimulants or atomoxetine. While high doses of quetiapine are sedative and are used to treat psychosis, low doses have a mildly sedative effect that can help reduce irritability and emotional liability associated with ADHD.

Unfortunately, there are insufficient studies of the treatment of ADHD in co-morbid cases, and offenders presenting with complex mix of co-morbidities. Our recommendations are therefore based on the experience of the authors, which are aligned with recommendations from guideline groups such as NICE [49]. In our experience, while a significant proportion of offenders with co-morbid conditions respond positively to the treatment of ADHD, there are cases that show limited or no response. Severe adverse effects on co-morbid conditions, including risk of psychosis, however appear to be extremely rare. The most common complaint is appetite loss. Overall, we conclude that while further work is needed to identify the predictors of good and poor response among patients with co-morbid conditions, the potential benefits of treatment outweigh the potential risks. The risks are minimised by careful monitoring of treatment effects during the titration phase of drug administration. In accordance with published guidelines [49], when titrating stimulants we recommend weekly assessments for 4–5 weeks, and less often for non-stimulants .

### Non-pharmacological treatments for offenders

Non-pharmacological treatments in the prison setting consist of psychological, educational, and occupational treatment programmes. These interventions should aim to: facilitate changes in life-long patterns of poor behavioural control, increase life satisfaction, build useful skills, and help the offender plan for civilian life after release. Mentorship programmes embedded in the treatment plan are likely to be additionally beneficial. Pharmacological treatment of ADHD symptoms will enable offenders who respond to such treatments to better engage in and benefit from non-pharmacological interventions in this setting. It is important to emphasize that, although ADHD medication can improve symptoms, concomitant non-pharmacological treatments are nearly always necessary to help the offender manage ADHD related problems and improve their behaviour.

#### Offender psychoeducation

There is a need to change common misconceptions and stereotypes about ADHD symptoms and treatments. Young people and adults find it helpful to understand that ADHD is a neurobiological disorder evident early in life and distinct from other behavioural problems. It is especially important for offenders to understand that although ADHD is pervasive, treatment may help improve self-control and level of function. In our experience prisoners value improvements in attention span and reduced levels of physical restlessness and emotional impulsivity that would enable them to benefit from education. Mental health professionals working with prisoners with ADHD should provide a clear explanation of ADHD symptoms, treatments, and expected outcomes, and educate the offender on the potential risks of remaining untreated or discontinuing treatment. Additionally, we recommend giving offenders an easy-to-read pamphlet that briefly highlights some facts about ADHD.

Offender psychoeducation is an integral part of intervention that should be initiated during imprisonment to increase its efficacy and to avoid overwhelming the offender upon release. We commonly observe that offenders are not adequately educated about their condition and take a passive role in their treatment plan. Furthermore, we have observed that an offender’s increased understanding of their condition helps them to engage in their pharmacologic and psychological treatment programmes and increase their sense of self-empowerment.

#### Psychological treatment programmes

Many UK prisons implement offending behaviour programmes that focus on addressing the risk of future offending behaviour, but these do not provide treatment for clinical conditions for offenders with ADHD. We recommend implementing a neurocognitive intervention that addresses offending behaviour and ADHD related and other behavioural co-morbid executive function deficits such as: difficulty with time-keeping, organizing, planning, and self-regulating emotions and behaviour.

Given the logistical limitations inherent in correctional institutions (e.g. restrictions on movement and variations in sentencing), it is important that treatment programmes are suitable and feasible for the prison environment. Appropriate programmes include those that can be: completed in a relatively short amount of time (less than 4 months), delivered in a small group setting with about 10 to 12 inmates once or twice per week, and administered to all offenders irrespective of age and gender.

To augment and fortify interventions we recommend providing the offender with personal support from a mentor (i.e. coach or champion). Prison staff, officers, substance abuse staff, primary and secondary care clinicians, educators, volunteers, and when appropriate even fellow prisoners, can be trained to provide one-on-one skill-building sessions. These one-on-one sessions emphasize a personal approach and can help the offender bridge lessons from the therapy room to daily life.

To the best of our knowledge the only psychological treatment programme developed to address antisocial behaviour and executive functioning deficits is Reasoning and Rehabilitation 2 ADHD (R&R2ADHD). R&R2ADHD is a treatment programme based on cognitive behaviour therapy designed to build pro-social competence [18] and may be used in non-offender and prison populations. It can be administered to all offenders irrespective of age and gender and completed in approximately 2 months. The programme’s short duration, comprised of 15 treatment sessions deliverable up to 2 times per week, makes it favourable to ensure completion. R&R2ADHD has an additional advantage of being suitable for both youth and adult offenders. Furthermore, mentorship is embedded within the programme — whereof an assigned coach or mentor meets one-on-one with the offender between sessions to help them consolidate and apply newly learned skills in their daily life.

While the evidence for R&R2ADHD efficacy is predominantly community based with a majority of male samples [19–21], results from a pilot trial at Her Majesty’s Prison Youth Offender Institution (HMP/ YOI) Feltham (a level 3 youth offender institution in the UK) indicated high rates of completion and universally positive feedback from enrolled youth offenders. We observed that the positive impact of R&R2ADHD on the youth offenders with ADHD at HMP/YOI Feltham was even more significant when prison staff were involved in the treatment programme. Oftentimes prison staff and officers have an established rapport with offenders, and involving them seems to improve offender engagement in the treatment programme. According to the 2013 London Mayor’s Office for Policing and Crime (MOPAC) report, R&R2ADHD was mentioned as an example of good practice and has received the full support of London prison governors and lead staff [52].

Other psychological approaches that may be helpful include cognitive remediation therapy (CRT) [53] and dialectical behavioural therapy (DBT) [54]. CRT applies techniques historically used to treat individuals with traumatic brain injury (e.g. deficits in planning, time management, and attention, impulse control). DBT was developed for the treatment of borderline personality disorder. Ideally, psychological interventions should take an eclectic approach drawing on these paradigms as well as cognitive behaviour therapy (CBT). The Young-Bramham Programme, is one such CBT intervention that can be used for adolescents and adults with ADHD [55].

#### Educational and occupational treatment programmes

Children with ADHD are at an increased risk of academic underachievement [56] and repeating an academic year [57], therefore all prisoners should have numeracy and literacy assessments to identify academic impairments. An appropriate individualized education plan based upon academic assessments developed by the prison education department can be additionally informed by mental health screen results, and previous mental health and school records, pending prisoner consent to information sharing between departments. The CHAT screening tool assesses for learning difficulties in young people (in the neurodisability section, part 5). These results should be used in developing a young offender’s education plan, and will consequently inform overall holistic care.

It is important that the education plan addresses gaps in the offenders’ academic core skills, focuses on strengths, includes ADHD support strategies, and is appropriate for those disengaged from the education system. Education support workers and volunteers from outside private organizations can be helpful with implementing the education plan. While education services have information pertinent to learning difficulties, they do not automatically liaise with mental health services. We have observed that problems with information sharing are primary barriers that need to be to overcome. Therefore, we recommend an ad hoc liaison between mental health and education services to ensure effective intervention.

Prison rules most often require that offenders complete an academic course (related to reading and writing) before participating in technical skill-building workshops. Because symptoms of inattention, hyperactivity, and behavioural difficulties can prevent the offender from meaningful participation in academic courses, this requirement is inherently biased against them. Imposing this requirement upon offenders with ADHD who are at risk of disengaging from and failing the academic course may result in extended prison time. For example, in England and Wales, if an offender fails the Imprisonment for Public Protection (IPP) course they are subject to an increased prison sentence.

We recommend waiving the requirement to complete an academic course and directing offenders towards educational and occupational programmes that suit their strengths (e.g. creative, technical, and/or athletic skills). Focusing on their strengths may not only reduce the occurrence of extended sentencing, but also the rate of offenders with ADHD in solitary confinement. In cases where solitary confinement has not been averted, we recommend shortening the period(s) of isolation and giving the offender an activity to occupy them while confined.

Participation in technical skill-building workshops can provide hands on experience and the opportunity to learn occupational and technical skills useful for life during and after prison. In our experience, maintenance jobs throughout the prison (e.g., housekeeping, kitchen, and garden work) that provide the opportunity to be physically active and occupied, are highly sought after by ADHD offenders.

In addition to acquiring technical skills, it is important that offenders are taught necessary personal life skills to equip them to successfully navigate civilian life after prison and not re-offend. Given the likelihood of executive function deficits, offenders with ADHD most often need help planning how to attain their goals. Long-term desires and goals (e.g., health, wealth, and happiness) need to be broken down into realistic achievable short-term plans and goals (e.g., self-care and employment). R&R2ADHD [18] notably includes a module that focuses on offender needs of this nature.

## Care management and multiagency liaison

As for people with mental health problems or related complex needs, offenders with ADHD require assistance from a wide variety of supportive services and agencies. It is important that these services are accessed and coordinated during imprisonment, not only to infer maximal benefit, but to ensure continuity of care once the prisoner is released. Supportive services and agencies, although distinct and separate entities, will need to liaise with each other to exchange information and help ensure comprehensive care. For example, education services should automatically be contacted when an offender with mental health issues is identified.

### Care plan and care coordination

In England people having complex needs are often eligible to receive a Care Programme Approach (CPA). A CPA is a structured multidisciplinary care management format designed to support people with severe and enduring mental health problems. Service users are allocated a care plan coordinator (e.g., community psychiatric nurse, social worker, psychologist or psychiatrist) who is responsible to review, access, and coordinate multiple available services on their behalf [58]. The structure of a CPA requires that issues are clearly addressed according to distinct domains of need (e.g., mental health, medication, accommodation, education, financial) and consequently clearly identifies which agencies are responsible to fulfil those needs. While the care plan coordinator is central to a CPA, it is important for them to encourage the service user to take an active role in their care plan as much as possible.

A CPA is routinely implemented for high risk patients in inpatient settings, yet less commonly in community mental health settings. Although the National Institute for Health and Clinical Excellence 2018 (NICE) guidelines [59] recommend that individuals with ADHD aged 16 and over receive a CPA, many offenders with ADHD are not adequately identified and subsequently do not receive an appropriate care management plan. In our experience, continual failure to properly identify offenders with ADHD and meet their needs can lead to some individuals to persist in offending behaviours and ‘fall through the gap’.

We recommend offenders with ADHD receive a CPA or similar care management plan and are assigned a care plan coordinator to oversee the plan. We also recommend implementing a medication management plan, which should be a core component of the care plan. A medication management plan addresses the need for regular built-in reviews by a suitably trained psychiatrist, and the need for monitoring medication adherence, which should be a key task of the care coordinator. When transitioning from youth to adult or between different institutions or out of prison, it is vital to maintain robust care coordination, continuity of care, and uninterrupted treatment with ADHD medication; using the CPA format can be effective in mitigating problems with service discontinuity that can arise during transitions. A sample CPA based on numerous ‘real life’ cases is provided in the supplementary material, see Additional file 1.

### Supportive services and agencies

The following people, services, and agencies may need to be accessed and coordinated according to the offenders’ individual needs:

- Parents and carers — adults responsible to provide general support at home and, where appropriate, give consent to treat
- Care plan coordinators — trained workers designated as the main point of contact responsible to develop a care plan and review, access, and coordinate appropriate available services
- Criminal justice services including diversion services — police, court, prison, and offender management unit staff responsible to move offenders through the offender pathway while safeguarding rights; an intermediary may be required
- Mental health services, including forensic mental health services — trained physicians, psychiatrists, psychologists, nurses, and therapists responsible to provide appropriate evaluations and treatments
- Primary and secondary care physicians — paediatricians, general practitioners, psychiatrists, and specialist physicians responsible to provide appropriate evaluations and treatments
- Social services — social workers responsible to access available financial support for appropriate healthcare, housing, and child care needs
- Educational services — teachers, therapists, nurses, and trained volunteers, responsible to provide appropriate evaluations and develop and implement individualized educational plans
- Mentorship programmes — trained coaches or champions responsible to provide one-on-one treatment or skill-building sessions
- Addiction services — trained clinicians, officers, or workers responsible to provide appropriate interventions and recovery support services
- Adoption and foster care agencies — trained workers responsible to access appropriate placements for youth offenders or children of offenders
- Probation services — trained officers responsible to supervise offenders and provide appropriate interventions
- Occupational, rehabilitation, and therapeutic services — trained workers, therapists, job coaches, and volunteers responsible to provide and/or access appropriate skill-building courses and employment opportunities to help the offender regain independence
- Immigration services — trained workers responsible to administrate and adjudicate cases of foreign offenders

### Support for female offenders

Despite women’s inclination to seek help, female offenders with ADHD may be less likely to be identified [12] and consequently less likely to receive effective support. As mentioned previously, pregnancy, responsibilities of parenting, and the risk of commonly prevalent co-morbid disorders complicate intervention and additionally cause female offenders to require varied and unique services. The immediate access of social and foster care services is crucial when an offender is pregnant and/or has child custody issues.

### Support for youth offenders

In developing a care plan for youth offenders it is important to involve parents or carers, whenever appropriate or possible. They can be helpful in supporting the youth once released, especially when it comes to medication compliance. Additionally, parents or carers may be required to give consent for treatment when the youth is under 18 years old, however, obtaining consent may be problematic in some cases. When the family is a part of the offenders’ problems or a family member is the victim, careful consideration is required before contacting and involving them. In addition to providing psychoeducation for the offender, families need to be educated on the facts about ADHD symptoms, treatments, and expected outcomes. The care plan coordinator should direct families to parent groups or other supportive organizations and resources to help them meet their child’s complex needs. The CHAT screening tool includes a care plan summary that highlights the young person’s needs. This information should be communicated to all professionals working with the young person in secure care, and to parents, carers, and care plan coordinators following release to ensure good continuity of care.

### Support for adult offenders

In developing a care plan for adult offenders it is important to encourage them to take an active role in their care management. When adults have been in the prison system for many years, it can be difficult to motivate them to engage in intervention and supportive services. We have observed that youth offender services and pathways are generally more developed and effective than those for adult offenders. Therefore, we recommend adult prisons adopt many of the same models and tools, such as mentorship programmes and comprehensive screens like the CHAT, which are used in youth offending institutions.

### Support upon prison release

Prison release and the transition into civilian life is a period of increased vulnerability requiring offenders with ADHD to receive specific timely support. There is a need for further research on ways to best support offenders with ADHD who are released from prison; meanwhile we recommend implementing a critical time intervention approach [60], in which a designated person meets with the offender just before and immediately after release from prison to help implement their care plan and ensure subsequent engagement in healthcare. Appropriate support would include: connecting the offender with their care plan coordinator, ensuring registration with a primary care physician, and helping them understand the implications of discontinuing treatment. Because uninterrupted treatment with ADHD medication is vitally important, we further emphasize the need to implement a medication management plan.

## Discussion

There is strong evidence of a high prevalence of offenders (both youth and adults, males and females) with ADHD in prison and who have increased risk of associated coexisting conditions and higher rates of recidivism. Paired with the evidence that treatment improves symptoms and outcomes, accurate identification and comprehensive treatment is warranted. Effective intervention is expected to have a positive impact on the offender and society and lead to increased productivity, decreased resource utilization, and most importantly reduced rates of re-offending.

The United Kingdom ADHD Partnership therefore hosted a meeting of experts on the topic who reviewed the literature and shared personal experiences. It was concluded that there were specific barriers within the prison that hindered the recognition of offenders within the system with ADHD. These include inadequate staff and offender awareness of ADHD symptoms and treatments; lack of training for mental health staff; inappropriate use of screening and diagnostic tools; inappropriate multimodal interventions, care management, supportive services, and multiagency liaison; and a lack of preparation for prison release to address the ongoing needs and care of prisoners with ADHD.

We successfully came to a consensus on practical ways to address these problems and it was clear that the work needs to commence with recognition and identification, and this will involve training. To appropriately care for the individual needs of each offender we recognize that a separate pathway for each mental health disorder (including ADHD) would need to be created. Realizing this may be a problem, we therefore aim to influence criminal justice systems to create a unifying mental health approach with different interventions that address each disorder. We envision a fully integrated intervention pathway—in which the information gathered from screenings and assessments is automatically shared with appropriate service departments for the immediate and coordinated implementation of necessary interventions.

The increased use of the CHAT screening tool, the launch of healthcare standards and commissioning guidance, and the development and implementation of the SECURE STAIRS (an integrated framework of care funded by the NHSE) together are supporting a unifying mental health approach within the secure estate for children and young people in England. Additionally, the NHSE’s report The Five Year Forward View for Mental Health [61] outlines general recommendations to support all offenders in the criminal justice system who are experiencing mental health problems. The report recommends expanding liaison and diversion schemes nationally and urges the establishment of comprehensive pathways and quality standards. We advise increasing the use of the B-BAARS [33, 35] as a screening tool to support the identification of adult offenders with ADHD. Our approach based upon expert consensus is directly aligned with these current efforts and offers practical solutions to address the un-met needs of offenders with ADHD.

The outcome of the review and consensus is detailed above and the key conclusions and recommendations that arose from it are briefly summarised in Table 1.

**Table 1:** Recommendations

*Identification and Assessment*	
1. Prison staff training to develop awareness of ADHD symptoms and co-morbid conditions (including how these may differ by age and gender), treatments, expected outcomes and the potential impact of prison regime on the offender with ADHD (e.g. greater risk of suicide, impact of segregation). This should include recognition that many offender mental health issues are secondary to ADHD.	
2. For youths, adoption of a suitable primary screen (e.g. CHAT) and a clinical diagnostic interview (e.g. ACE). If a rating scale is given (e.g. SNAP-IV, CBRS) this should be sensitive to both inattention and hyperactivity/impulsivity symptoms.	
3. For adults, adoption of a suitable primary screen (e.g. B-BAARS) and a clinical diagnostic interview (e.g. ACE+, CAADID, DIVA-2). If a rating scale is given (e.g. BAARS) this should be sensitive to both inattention and hyperactivity/impulsivity symptoms.	
*Interventions and Treatment*	
4. All treatments should include psychoeducation about ADHD, including symptoms, co-morbidity, pharmacological and non-pharmacological treatments, side-effects of treatment and expected outcomes.	
5. Adoption of appropriate pharmacological and non-pharmacological treatments (see Fig. 1).	
6. Adoption of appropriate educational and occupational programmes designed to increase engagement (see Fig. 1).	
7. Educational and occupational programmes should be prioritised that advance vocational, creative, technical, and/or athletic skills.	
*Care Management and Multiagency Liaison*	
8. There should be close liaison between education and mental health services within the criminal justice system	
9. A care plan coordinator should be assigned to the offender while in prison.	
10. A comprehensive care plan should be established, including a medication management plan, for the offender while in prison (see Additional file 1, online supplementary material).	
11. The care plan should also plan to prepare the offender with ADHD for release from prison (e.g. effecting a seamless transition to ensure continuity of care and uninterrupted treatment with ADHD medication; arranging appropriate links with supportive services and agencies).	
12. A critical time intervention approach should be established for a designated person to support the offender through the release process, support implementation of the care plan and ensure engagement in healthcare.	

While a practical approach to effectively identify and treat offenders with ADHD was achieved at the meeting, it was clear that future research is needed to identify optimal clinical operating models and monitor their implementation and measure their success. It will be valuable to investigate the impact of accurate identification and specifically targeted multimodal treatments on offender health, behaviour, and offence related outcomes. Further research on the needs of female offenders is needed.

A recent study investigating the economic consequences of ADHD in prison has conservatively estimated that the financial burdon of medical and behaviour-related prison care is £11.7 million per annum [62] and future research should evaluate the financial benefits to society of effectively treating offenders with ADHD (compared with not-treating) using functional related outcomes. It will be essential to demonstrate the cost effectiveness of intervention using health economic modelling techniques to garner governmental support and effect change in criminal justice and mental health service policies.

## Conclusion

This consensus will inform effective identification and treatment of offenders with ADHD. Appropriate intervention is expected to have a positive impact on the offender and society and may lead to increased productivity, decreased resource utilization, and most importantly reduced rates of re-offending.

## Additional file

Additional file 1: Care Programme Approach (CPA) report. This is not an actual CPA, but is a sample CPA report based on ‘real life’ cases. (DOCX 12 kb)
