# Supplementary material for: Identification and treatment of offenders with attention-deficit/hyperactivity disorder in the prison population: a practical approach based upon expert consensus
Source: BMC Psychiatry. 2018 Sep 4;18:281. doi: 10.1186/s12888-018-1858-9 (PMC6122636; doi:10.1186/s12888-018-1858-9)
Supplement: Supplementary file 1 — Care Programme Approach (CPA) report. This is not an actual CPA, but is a sample CPA report based on ‘real life’ cases. (DOCX 12 kb) [file 12888_2018_1858_MOESM1_ESM.docx]

**Sample of a Care Programme Approach (CPA) Report**

**Introduction**

A 21-year old man, who is on remand (pre-trial) in prison, has been charged with common assault and theft. He has a known history of ADHD, having previously been referred to, and assessed by, specialist community services. He has a history of variable engagement with healthcare providers, and of intermittent non-adherence to treatment. He has a history of earlier co-morbid conduct disorder, and has been prescribed methylphenidate in accordance with NICE guidelines.

**Engagement**

He has been managed under CPA for 2 years, and his last CPA review took place eight months ago. The process has been complicated by non-attendance and non-adherence to treatment. He has attended 50% of recommended monthly reviews, but has participated well during these sessions. Psychoeducation has been a major focus of the work with him, encouraging adherence by working to remove potential barriers (e.g. recent move of accommodation with the potential for treatment dislocation).

**Content of sessions**

Receiving ongoing assessment and management of his ADHD symptoms, and psychoeducation to improve understanding and awareness of ADHD and promote adherence. Liaison has taken place between community and prison-based services to ensure appropriate information handover and continuity of care. He may require additional support because this is his first time in an adult prison in which bullying occurs commonly and illegal substances are freely available.

**Formulation**

Has history of ADHD with co-morbid conduct disorder.

Care and treatment have been complicated by intermittent non-engagement and non-adherence.

Recently sentenced to an adult prison. This introduces a potential new stressor indicating a need for enhanced support, at least for an initial period.

**Summary and recommendations**

1.     Continue to offer care coordination with an identified clinician during his time in prison

2.     Continue to prescribe and monitor the effects of methylphenidate, including any emerging side effects

3.     Continue to offer psychological support, with a focus on psychoeducation, engagement, adherence and the removal of potential barriers

4.     Use CPA processes as an ongoing framework for his care and management

5.     Liaise with relevant community services to ensure appropriate information receipt and transfer, and continuity of care
